# Association between triglycerides, known risk SNVs and conserved rare variation in *SLC25A40* in a multi-ancestry cohort

**DOI:** 10.1186/s12920-020-00854-2

**Published:** 2021-01-06

**Authors:** Elisabeth A. Rosenthal, David R. Crosslin, Adam S. Gordon, David S. Carrell, Ian B. Stanaway, Eric B. Larson, Jane Grafton, Wei-Qi Wei, Joshua C. Denny, Qi-Ping Feng, Amy S. Shah, Amy C. Sturm, Marylyn D. Ritchie, Jennifer A. Pacheco, Hakon Hakonarson, Laura J. Rasmussen-Torvik, John J. Connolly, Xiao Fan, Maya Safarova, Iftikhar J. Kullo, Gail P. Jarvik

**Affiliations:** 1 Division of Medical Genetics, School of Medicine, University of Washington Medical Center, 1705 NE Pacific St, Box 357720, Seattle, WA 98195 USA; 2 Department of Biomedical Informatics Medical Education, School of Medicine, University of Washington, Seattle, WA USA; 3 Center for Genetic Medicine, Feinberg School of Medicine, Northwestern University, Chicago, IL USA; 4 Kaiser Permanente Washington Health Research Institute, Seattle, WA USA; 5 Department of Biomedical Informatics, Vanderbilt University Medical Center, Nashville, TN USA; 6 Department of Medicine, Vanderbilt University Medical Center, Nashville, TN USA; 7 Division of Endocrinology, Department of Pediatrics, Cincinnati Children’s Hospital and the University of Cincinnati, Cincinnati, OH USA; 8 Genomic Medicine Institute, Geisinger, Danville, PA 17822 USA; 9 Department of Genetics, University of Pennsylvania, Philadelphia, PA USA; 10 Center for Applied Genomics, The Children’s Hospital of Philadelphia, Philadelphia, PA USA; 11 Department of Preventive Medicine, Northwestern University Feinberg School of Medicine, Chicago, IL USA; 12 Department of Cardiovascular Medicine, Mayo Clinic, Rochester, MN USA; 13 Gonda Vascular Center, Mayo Clinic, Rochester, MN USA; 14 Department of Genome Sciences, University of Washington, Seattle, WA USA

**Keywords:** Genetics, Cardiovascular disease, Triglycerides, Electronic health records

## Abstract

**Background:**

Elevated triglycerides (TG) are associated with, and may be causal for, cardiovascular disease (CVD), and co-morbidities such as type II diabetes and metabolic syndrome. Pathogenic variants in *APOA5* and *APOC3* as well as risk SNVs in other genes [*APOE* (rs429358, rs7412), *APOA1/C3/A4/A5* gene cluster (rs964184), *INSR* (rs7248104), *CETP* (rs7205804), *GCKR* (rs1260326)] have been shown to affect TG levels. Knowledge of genetic causes for elevated TG may lead to early intervention and targeted treatment for CVD. We previously identified linkage and association of a rare, highly conserved missense variant in *SLC25A40*, rs762174003, with hypertriglyceridemia (HTG) in a single large family, and replicated this association with rare, highly conserved missense variants in a European American and African American sample.

**Methods:**

Here, we analyzed a longitudinal mixed-ancestry cohort (European, African and Asian ancestry, N = 8966) from the Electronic Medical Record and Genomics (eMERGE) Network. We tested associations between median TG and the genes of interest, using linear regression, adjusting for sex, median age, median BMI, and the first two principal components of ancestry.

**Results:**

We replicated the association between TG and *APOC3*, *APOA5*, and risk variation at *APOE*, *APOA1/C3/A4/A5* gene cluster, and *GCKR*. We failed to replicate the association between rare, highly conserved variation at *SLC25A40* and TG, as well as for risk variation at *INSR* and *CETP*.

**Conclusions:**

Analysis using data from electronic health records presents challenges that need to be overcome. Although large amounts of genotype data is becoming increasingly accessible, usable phenotype data can be challenging to obtain. We were able to replicate known, strong associations, but were unable to replicate moderate associations due to the limited sample size and missing drug information.

## Background

Triglyceride (TG) levels are a marker for health outcomes. High TG, (defined as 200 < TG < 500 mg/dL in adults), are associated with, and may cause, cardiovascular disease (CVD) [1-4]. High TG is also associated with type II diabetes and metabolic syndrome [5, 6]. TG levels are affected by environmental variables such as diet, alcohol consumption, smoking, and exercise [7-11]. Maintaining healthy levels of TGs may improve health outcomes [6, 12].

Both hypo- and hypertriglyceridemia (HTG) have major genetic components such as heterozygous variation at *APOC3* and *APOA5*, respectively [13-15]. More recently, homozygous and compound heterozygous null variation at *ANGPTL3* and *ANGTPL4* have been shown to be associated with low TG [16, 17]. SNVs in the genes *APOE*, *GCKR*, *APOA1/C3/A4/A5* gene cluster, *INSR* and *CETP* have smaller effects on TG [18-20].

We previously identified linkage and association of a rare, highly conserved missense SNV, rs762174003, in *SLC25A40* with HTG in a large family, and replicated the association with rare missense SNVs at highly conserved sites in *SLC25A40* in a European American (EA) and African American (AA) sample [21]. *SLC25A40* encodes a mitochondrial transmembrane protein [22]. In drosophila, *SLC25A40* loss-of-function was shown to contribute to mitochondrial damage due to excessive oxidation [23]. Here, we attempt to replicate the association between deleterious variation at *SLC25A40,* as well as other reported associations with variation at *APOE, GCKR, APOA1/C3/A4/A5* gene cluster*, INSR,* and *CETP,* with TG in a multi-ancestry population from the Electronic Medical Records and Genomics (eMERGE) Network phase 3 study [24, 25].

## Methods

### Participants and phenotype data

Participants were ascertained from each of nine phase 3 eMERGE sites, a study tasked with investigating return of results from the sequencing of a panel of ~ 100 genes in a large (N = 25,000), diverse cohort of participants [24]. Participants ages 18 years and older were included, as the distribution for TG levels in children differs from that of adults. We ascertained relevant patient data from the clinical records, including TG level measured in mg/dL (N = 12,229 adult participants), age, sex, weight, height and body mass index (BMI), measured at the same clinical visit. We also ascertained relevant ICD9 and ICD10 codes for lipidemias, pancreatic cancer, and pancreatitis. Thirteen participants with chylomicronemia (ICD9 code 272.3, ICD10 code E78.3), 12 participants with Kaposi’s sarcoma (ICD9 code 176 and ICD10 code C46) and 2,539 participants with morbid obesity (ICD9 code 278.01 and ICD10 code E66.01) or bariatric surgery were removed as their TG values are likely associated with unaccounted for exposures. Quality control was performed within participants as well. Participants who had maximum TG (maxTG) < 40 and had more than one record verifying this low TG were retained. However, one participant who had a single record with TG = 19 mg/dL was removed. Records within 2 weeks prior or 4 weeks after a diagnosis of pancreatitis (ICD9 code 577.0, ICD10 code K85.9) for an individual were removed (382 participants), as high TG are correlated with pancreatitis and is likely due to an environmental exposure such as alcohol. Similarly, individual level records coincident and after a diagnosis of pancreatic cancer were removed (147 participants). Quality control of BMI was performed using height and weight data as described by Goodloe et. al [26]. For participants with only BMI data (i.e., no height or weight data), values more than 5 standard deviations from the participant specific median BMI were removed.

### Genotype data

A subset of individuals (N = 24,956) in eMERGE were genotyped using a custom capture target containing sequence for 788 genes with partial to full exon coverage, including 58 of the 59 genes considered actionable by the American College of Medical Genetics (ACMG) [27, 28], and detects 62,051 SNVs. Sequencing was performed at two sites: Partners Healthcare Laboratory for Molecular Medicine (LMM) and Baylor. Samples from dropped out participants, that failed quality control or had sex discrepancy were removed (N = 229). After removing duplicate data for a single participant, sequence data for all participants was aligned to a merged target sequence map, using the Burrows-Wheeler Aligner version 0.7.10 [29]. Genotypes were jointly called on all participants using the Genome Analysis Toolkit version 3.5 [30]. 896 Variants with genotype missingness rate > 5% were removed. Individual level genotyping rate was > 98% for all participants. After cleaning the data, 8970 participants with both phenotype and genotype data remained in the analysis. Four additional participants were removed due to their influence on the residual model (see results).

Principal components (PC) of ancestry was calculated on a pruned set of 1571 SNVs (r^2^ < 0.7, MAF > 0.05). Self-reported ancestry aligned well with the first two PCs.

Exon sequence data was available for the relevant genes *SLC25A40*, *APOC3*, and *APOA5*. Two individuals with known pathogenic variants in *APOA5* (rs201079485, rs147528707) were removed from analysis. Exon sequence data was also available for known TG risk SNVs rs429358 and rs7412 (which determine the *APOE* ε2/3/4 genotype), rs7248104 (*INSR*), rs1260326 (*GCKR*), rs7205804 (*CETP*), and rs964184 (*APOA1/C3/A4/A5* gene cluster).

### Analysis

The phenotype of interest, medTGres, was calculated as the residuals from the median log10 transformed TG for each individual, adjusted for median age, sex, median BMI, presence of hypertriglyceridemia (HTG, ICD9 code 272.1 and ICD10 code E78.1), presence of hyperlipidemia (HL, ICD9 codes 272.2, 272.4, 272.9 and ICD10 codes E78.2, E78.4, E78.9), *APOE* genotype, known risk SNVs, known *APOC3* protective SNVs, the first two principal components (PCs) of ancestry, and site. The variable for median age was centered around age 50, as this is when TG levels tend to descend, and then squared, as the relationship between TG levels and age is parabolic. We log10 transformed TG as the distribution is highly skewed and the transformation makes it nearly Normal. We used the median transformed TG (medTG) value over all records for each individual because the median protects against outliers that could be due to non-fasting measurements. The resulting residuals from the above linear model (medTGres) were used as the primary phenotype. We also used a similar residual from the adjusted maximum log10 transformed TG (maxTGres), as using the maximum protects against errors introduced from treatment with Niacin, fibrates or binders for high TG. Given our more complete local data from the University of Washington and Kaiser Permanente Washington (UWKP) site, we expect about 3% of the participants to be receiving such treatment at some time point in their clinical records [31]. However, this data was not available to us study-wide.

In addition to assessing the significance of the known risk variation, we analyzed variants in *SLC25A40* that are likely to affect TG levels [21]. This included rare (maximum population MAF < 0.005) variants that either caused an early termination in the first 90% of the coding region, or changed the amino acid sequence. Missense variants were further constrained to be evolutionarily conserved (Genomic Evolutionary Rate Profiling (GERP) score > 4.8) [32]. The maximum population MAF was derived from populations in the gnomAD database that have not experienced a bottleneck (i.e., ignored MAF from Ashkenazi Jewish or Finnish populations) [33, 34]. We performed four separate gene-wise tests for association with medTGres and maxTGres using 1. Genotype at single SNVs with more than 10 heterozygotes, 2. All other SNVs collapsed into a single indicator variable, 3. All other rare missense SNVs collapsed into a single indicator variable and 4. All other early terminations collapsed into a single indicator variable.

## Results

### Demographics

**Fig. 1:**
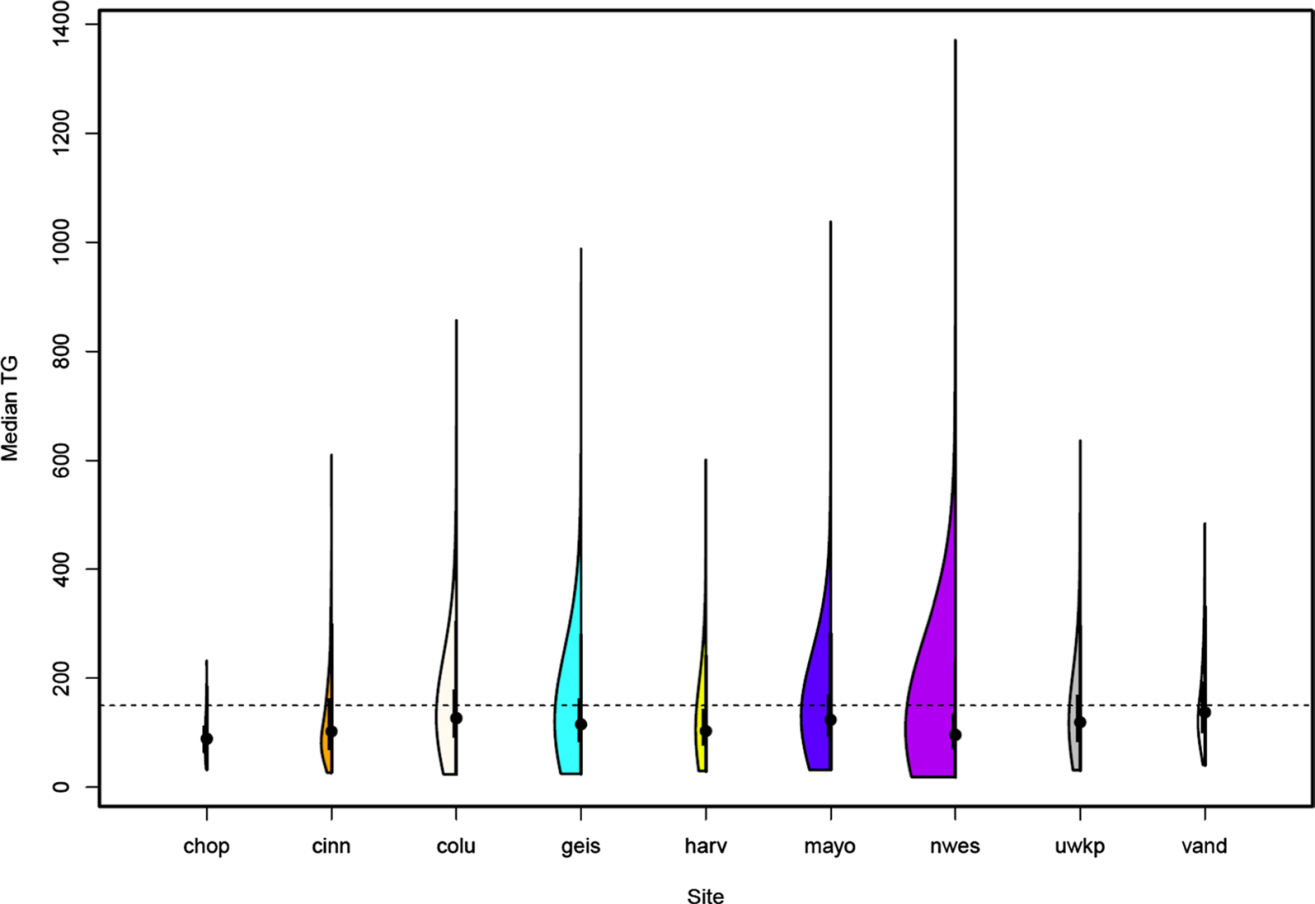
Distribution of median triglyceride by site. The horizontal line at 150 mg/dL represents the maximum normal level for an adult

**Fig. 2:**
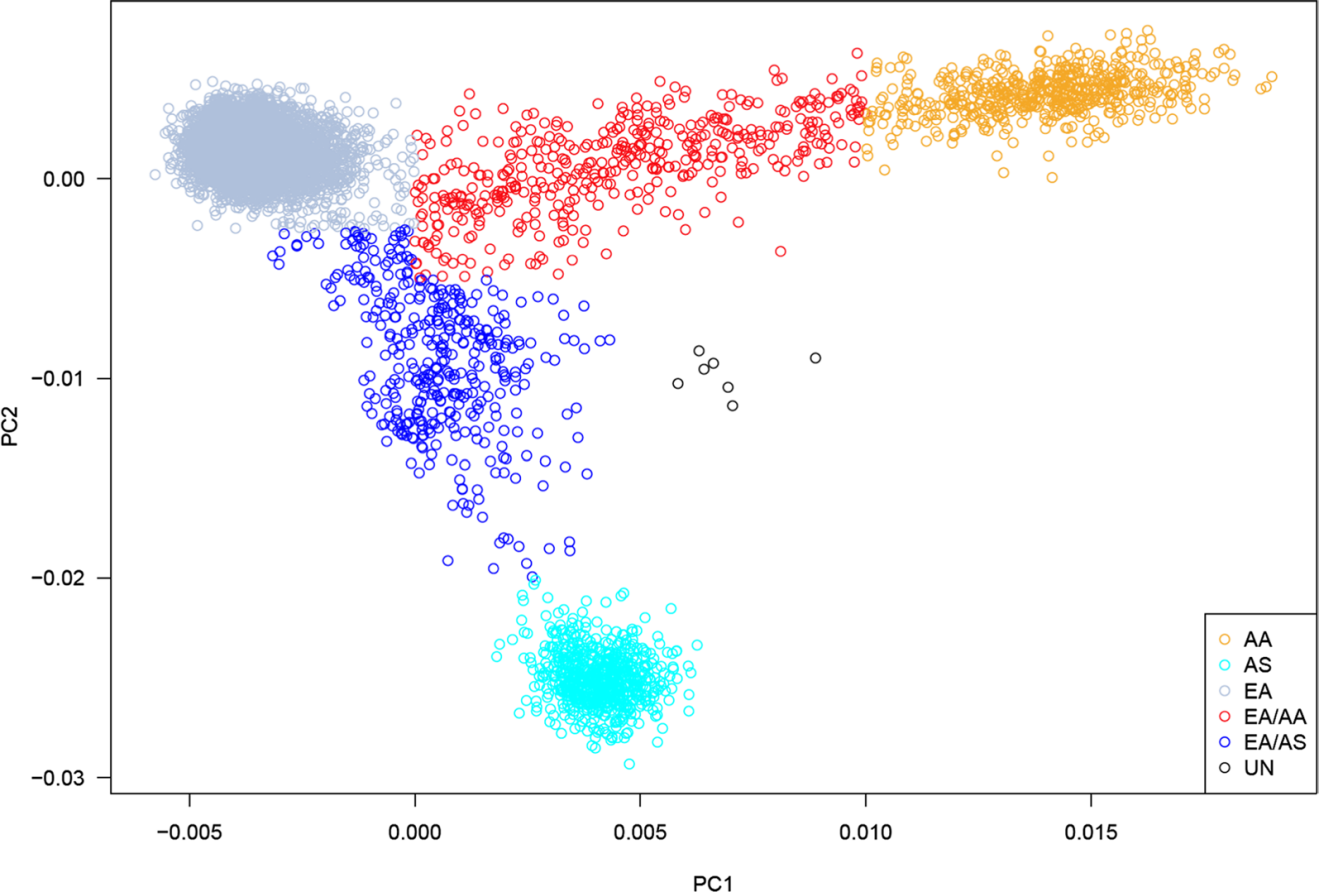
First and second principal components of ancestry. *AS* Asian ancestry, *EA* European ancestry, *AA* African ancestry, *UN* unknown ancestry

Demographics of the participants included in these analyses are given in Table 1. There were slightly more females than males at most sites. The distribution of age varied widely among the sites, with younger participants from Children’s Hospital of Philadelphia (CHOP) and Cincinnati Children’s Hospital Medical Center. Median BMI tended to be above normal (> 25), but there were some participants with low BMI (< 18.5) (Columbia, Mayo). There were 51 participants with very low median BMI < 11, all from Mayo, with at least 3 records supporting this measurement. The median TG ranged between 19 and 1371 mg/dL over all participants, but the majority of participants had median TG < 150 mg/dL, which is defined as normal (Fig. 1). The number of TG records available per participant varied widely, with a median of 6 overall. Most participants were of European ancestry (77%) followed by African ancestry (7%) and Asian ancestry (6%) (Table 2, Fig. 2). Most of the participants of Asian ancestry (82%) were from UWKP and most of the participants of African ancestry (62%) were from Northwestern and Cincinnati.

**Table 1:** Demographics by site

Site	Total	Nfemale (%F)	Median age Avg (min, max)	Median BMI^a^ Avg (min,max)	Median TG^b^ Avg (min, max)	Number of records	
						Median	Max
CHOP	35	15 (43)	19 (18, 21)	27 (17, 48)	94 (33, 232)	1	4
Cinc	287	150 (52)	21 (18, 56)	27 (14, 51)	130 (27, 610)	2	58
Colu	618	341 (55)	50 (18, 87)	27 (11, 45)	146 (24, 857)	5	105
Geis	1278	736 (58)	49 (18, 89)	30 (16, 58)	134 (25, 988)	5	52
Harv	932	533 (57)	53 (18, 89)	27 (15, 45)	121 (30, 601)	7	169
Mayo	2023	1140 (56)	53 (21, 69)	27 (9, 45)	139 (32, 1038)	15	109
Nwes	1962	1068 (54)	59 (18, 89)	28 (15, 52)	116 (19, 1371)	4	57
UWKP	1685	955 (57)	57 (22, 89)	27 (15, 40)	137 (32, 636)	5	48
Vand	146	50 (34)	64 (26, 86)	29 (18, 47)	157 (41, 484)	12.5	78
Total	8966	4988 (56)	53 (18, 89)	28 (9, 58)	131 (19, 1371)	6	169

*CHOP* Children’s hospital of Philadelphia, *Cinc* Cincinnati, *Colu* Columbia, *Geis* Geisinger, *Harv* Harvard, *Mayo* Mayo Clinic, *Nwes* Northwestern, *UWKP* University of Washington and Kaiser Permanente Washington, *Vand* Vanderbilt

^a^Low BMI, and the associated heights and weights were manually checked for consistency within an individual, across records

^b^Low TG was manually checked for consistency within an individual, across records

**Table 2:** Distribution of ancestry by site

Site	AA	AS	EA	EA/AA	EA/AS	UN
CHOP	17	1	16	0	1	0
Cinc	124	0	139	21	3	0
Colu	88	29	238	215	48	0
Geis	28	4	1200	30	16	0
Harv	46	18	766	68	34	0
Mayo	5	14	1989	1	14	0
Nwes	213	50	1512	84	101	2
UWKP	23	539	933	19	166	5
Vand	2	0	143	0	1	0
All	546	655	6936	438	384	7

Principal components of ancestry are given in Fig. 2

*Chop* Children’s hospital of Philadelphia, *Cinc* Cincinnati, *Colu* Columbia, *Geis* Geisinger, *Harv* Harvard, *Mayo* Mayo Clinic, *news* Northwestern, *UWKP* University of Washington and Kaiser Permanente Washington, *Vand* Vanderbilt, *AA* African American, *AS* Asian American, *EA* European American, *UN* unknown

### RiskSNVs

Genotype counts for *APOE* and risk SNVs at *APOC3*, *APOA5*, *APOA1/C3/A4/A5* gene cluster and *GCKR* are given in Tables 3 and 4. The distribution of *APOE* genotypes follows expectations, given known allele frequencies. Sixty-two participants were heterozygous for 1 of 4 protective *APOC3* SNVs (rs76353203, rs147210663, rs138326449, rs140621530). Eighty-three participants had at least one of two risk alleles at *APOA5* SNV rs2075291.

**Table 3:** *APOE* genotypes counts and percentages derived from rs429358 and rs7412, by site and ancestry

Site	ε2/ε2	ε2/ε3	ε2/ε4	ε3/ε3	ε3/ε4	ε4/ε4
CHOP	0	5 (14)	0	19 (54)	10 (29)	1 (2.9)
Cinc	2 (0.7)	41 (14)	5 (1.7)	146 (51)	79 (28)	14 (4.9)
Colu	3 (0.5)	73 (12)	13 (2.1)	373 (60)	143 (23)	13 (2.1)
Geis	15 (1.2)	171 (13)	27 (2.1)	769 (60)	281 (22)	15 (1.2)
Harv	1 (0.1)	120 (13)	30 (3.2)	561 (60)	195 (21)	25 (2.7)
Mayo	14 (0.7)	180 (9)	41 (2)	1170 (58)	560 (28)	58 (2.9)
Nwes	8 (0.41)	227 (12)	51 (2.6)	1189 (61)	451 (23)	36 (1.8)
UWKP	10 (0.59)	206 (12)	22 (1.3)	1059 (62)	370 (22)	18 (1.1)
Vand	4 (2.7)	18 (12)	6 (4.1)	77 (53)	34 (23)	7 (4.8)
*Ancestry*						
AA	3 (0.55)	93 (17)	24 (4.4)	235 (43)	167 (31)	24 (4.4)
AS	1 (0.15)	87 (13)	4 (0.61)	444 (68)	116 (18)	3 (0.46)
EA	49 (0.71)	776 (11)	152 (2.2)	4157 (60)	1658 (24)	144 (2.1)
EA/AA	4 (0.91)	45 (10)	14 (3.2)	255 (58)	105 (24)	15 (3.4)
EA/AS	0	39 (10)	1 (0.26)	268 (70)	75 (19.5)	1 (0.26)
UN	0	1 (14)	0	4 (57)	2 (29)	0
Total	57 (0.64)	1041 (12)	195 (2.1)	5363 (60)	2123 (24)	187 (2.1)

*Chop* Children’s hospital of Philadelphia, *Cinc* Cincinnati, *Colu* Columbia, *Geis* Geisinger, *Harv* Harvard, *Mayo* Mayo Clinic, *news* Northwestern, *UWKP* University of Washington and Kaiser Permanente Washington, *Vand* Vanderbilt, *AA* African American, *AS* Asian American, *EA* European American, *UN* unknown

**Table 4:** Risk SNV genotype counts by site and ancestry

Site	*APOC3* 01	^†^*APOA5* 00/01/11/02	*APOA1*/*C3*/*A4*/*A5* 00/01/11	*GCKR* 00/01/11	*INSR* 00/01/11	*CETP* 00/01/11
CHOP	0	35/0/0/0	31/4/0	15/16/4	4/16/15	19/12/4
Cinc	3	286/1/0/0	183/89/15	156/94/37	37/94/156	151/91/45
Colu	8	611/7/0/0	416/180/22	276/263/79	79/263/276	300/238/80
Geis	10	1273/4/0/1	924/317/37	414/640/224	224/640/414	412/640/226
Harv	11	932/0/0/0	626/277/29	310/451/171	171/451/310	316/450/166
Mayo	3	2021/2/0/0	1435/537/51	695/982/346	346/982/695	642/989/392
Nwes	22	1951/9/2/0	1360/546/56	746/905/311	311/905/746	742/922/298
UWKP	5	1628/54/3/0	1132/487/66	607/764/314	314/764/607	637/809/239
Vand	0	146/0/0/0	110/34/2	53/64/29	29/64/53	36/77/33
*Ancestry*						
AA	7	542/4/0/0	343/183/20	269/227/50	13/120/413	407/129/10
AS	0	595/55/5/0	376/245/34	306/274/75	149/322/184	315/286/54
EA	53	6930/5/0/1	5006/1752/178	2362/3396/1178	1271/3384/2281	2181/3440/1315
EA/AA	2	438/0/0/0	278/143/17	188/201/49	38/176/224	218/180/40
EA/AS	0	371/13/0/0	208/147/29	156/165/63	44/172/168	131/190/63
UN	0	7/0/0/0	6/1/0	3/4/0	0/5/2	3/3/1
Total	62	8883/77/5/1	6217/2471/278	3272/4179/1515	1515/4179/3272	3255/4228/1483

*APOC3* consists of 4 SNVs (rs76353203, rs147210663, rs138326449, rs140621530), *APOA5* = rs2075291, *APOA1/C3/A4/A5 gene cluster* = rs964184, *GCKR* = rs1260326. *INSR* = rs1260326, *CETP* = rs7205804

*Chop* Children’s hospital of Philadelphia, *Cinc* Cincinnati, *Colu* Columbia, *Geis* Geisinger, *Harv* Harvard, *Mayo* Mayo Clinic, *news* Northwestern, *UWKP* University of Washington and Kaiser Permanente Washington, *Vand* Vanderbilt, *AA* African American, *AS* Asian American, *EA* European American, *UN* unknown

00 = common genotype, 01 = heterozygote, 11 = alternate homozygote,

^†^rs2075291 has three alleles. For this SNV, 02 = rare heterozygote (Allele 2 has frequency less than allele 1)

### *SLC25A40* genotype data

Twenty-one rare SNVs, consisting of 2 stop gains, 3 frame shifts and 16 evolutionarily conserved missense variants, were heterozygous in the data set for 32 participants (Table 5). There were 119 participants who were heterozygous at the missense rs724665 and this SNV is considered separately from the others. Each participant was heterozygous for at most one of the SNVs. Three participants were heterozygous at the frame shifts and 2 participants were heterozygous at the stop gains, limiting the power of the tests.

**Table 5:** *SLC25A40* variants which are rare and cause a coding change

rsID	POS	REF	ALT	Annotation	GERP	N	Ancestry
rs943387265	87466057	C	T	MS	5.29	1	AS
NA	87470950	T	A	MS	5.82	1	EA
rs1022111508	87470978	T	C	MS	5.92	1	AA
rs369745713	87470986	T	C	MS	5.92	2	EA
rs148648460	87473070	G	C	MS	5.67	1	EA
rs140104130	87473143	A	T	MS	5.67	1	EA
NA	87473157	T	TGTCTAAGTATTTTC	FS	NA	1	EA/AA
rs746455065	87473158	CAT	C	FS	NA	1	EA
rs147753823	87473175	A	C	MS	5.67	6	EA
rs775550958	87476272	G	A	MS	5.54	1	EA
rs1035790230	87476319	C	T	SG	5.54	1	EA
rs890753675	87476428	A	G	MS	5.54	1	AA
NA	87477248	T	C	MS	5.13	1	EA
rs724665^a^	87477257	G	A	MS	5.13	119	AA (4), EA (112), EA/AS (3)
rs200954020	87479214	C	T	MS	5.48	6	AS (5), EA/AS (1)
rs1021091982	87483577	C	T	MS	5.91	1	EA
rs748627166	87483582	A	T	SG	3.57	1	EA
rs1443219471	87483607	C	G	MS	5.03	1	EA
NA	87487954	G	A	MS	5.43	1	EA
NA	87488022	TC	T	FS	NA	1	EA/AS
rs747192743	87488041	A	G	MS	5.53	2	AS, EA

POS = position on human build hg19 chromosome 7, REF = reference allele, ALT = alternative allele, MS = missense, FS = frameshift, SG = stop gain, AS = Asian ancestry, EA = European Ancestry, AA = African ancestry

^a^rs724665 was assessed separately from the other missense variants

### Residual model

The final residual model, containing only significant covariates is shown in Table 6. Although centered-median-age-squared was significantly associated with log10(medTG), its effect is near zero (*β* = − 4e−05). Males had higher log10(medTG) (*β* = 0.02). Increased BMI was associated with higher log10(medTG) (*β* = 0.01). Similarly, individuals with HTG or HL had higher log10(medTG) (*β* = 0.3 and 0.08, respectively) than other participants. SITE was also highly associated with log10(medTG) (F-test *p* < 2e−16). Neither the *INSR* or *CETP* SNVs were significantly associated with median or maximum TG (*p* > 0.16), and were not included in the model (data not shown). Four potentially influential outlying participants (as defined by their cook’s distance [35]) in either model (medTGres or maxTGres) were removed, leaving a total of 8,966 participants with complete phenotype and genotype data for analysis. None of the participants with low BMI (median BMI < 15) appeared to influence the models.

**Table 6:** Effect of covariates in linear model used to adjust median TG

Variable	*β*	p
Age	−4.04e−05	2.15e−07
Sex (Male)	0.02	3.08e−09
BMI	0.01	2.46e−132
*APOE* (rs429358)	0.01	1.98e−03
*APOE* (rs7412)	0.04	1.65e−11
*GCKR* (rs1260326)	0.03	1.23e−27
*APOA1/C3/A4/A5* gene cluster (rs964184)	0.04	1.58e−29
*APOC3*	−0.23	1.32e−21
*APOA5* (rs2075291)	0.09	2.65e−05
PC1	−2.84	2.57e−09
PC2	−2.17	8.62e−11
SITE	NA	< 2e−16
HTG	0.3	3.34e−176
HL	0.08	6.92e−72

Age = centered-median-age-squared, BMI = median BMI, SITE = indicators for each site, HTG = hypertriglyceridemia, HL = unspecified or mixed hyperlipidemia and other unspecified disorders of lipid metabolism. Participant counts for HL exclude participants with HTG. PC = Principal component of ancestry. *APOC3* is an indicator for genotype at 4 SNVs (rs76353203, rs147210663, rs138326449, rs140621530)

### Association with known TG risk SNVs

Previously published risk SNVs at *APOE*, *GCKR*, *APOA5* and *APOC3* were associated with TG (Table 6). The minor alleles at the *APOE* SNVs rs429358 and rs7412 were associated with increased log10(medTG) (*β* = 0.01 and 0.04, respectively) and are jointly significantly associated with log10(medTG) (F-test *p* = 1.1e−11). Under an additive model, the minor alleles at *GCKR* and *APOA1/C3/A4/A5* gene cluster were also significantly positively associated with log10(medTG) (*GCKR*: *β* = 0.03, *p* < 2e−16, *APOA1/C3/A4/A5* gene cluster: *β* = 0.04, *p* < 2e−16). Under a dominant model, presence of either minor allele at *APOA5* was positively associated with log10(medTG) (*β* = 0.09, *p* = 2.7e−05). Jointly, a single copy of any of the minor alleles at the four *APOC3* SNVs were negatively associated with log10(medTG) (*β* = -0.23, *p* = 1.3e−21).

### Primary analysis

**Fig. 3:**
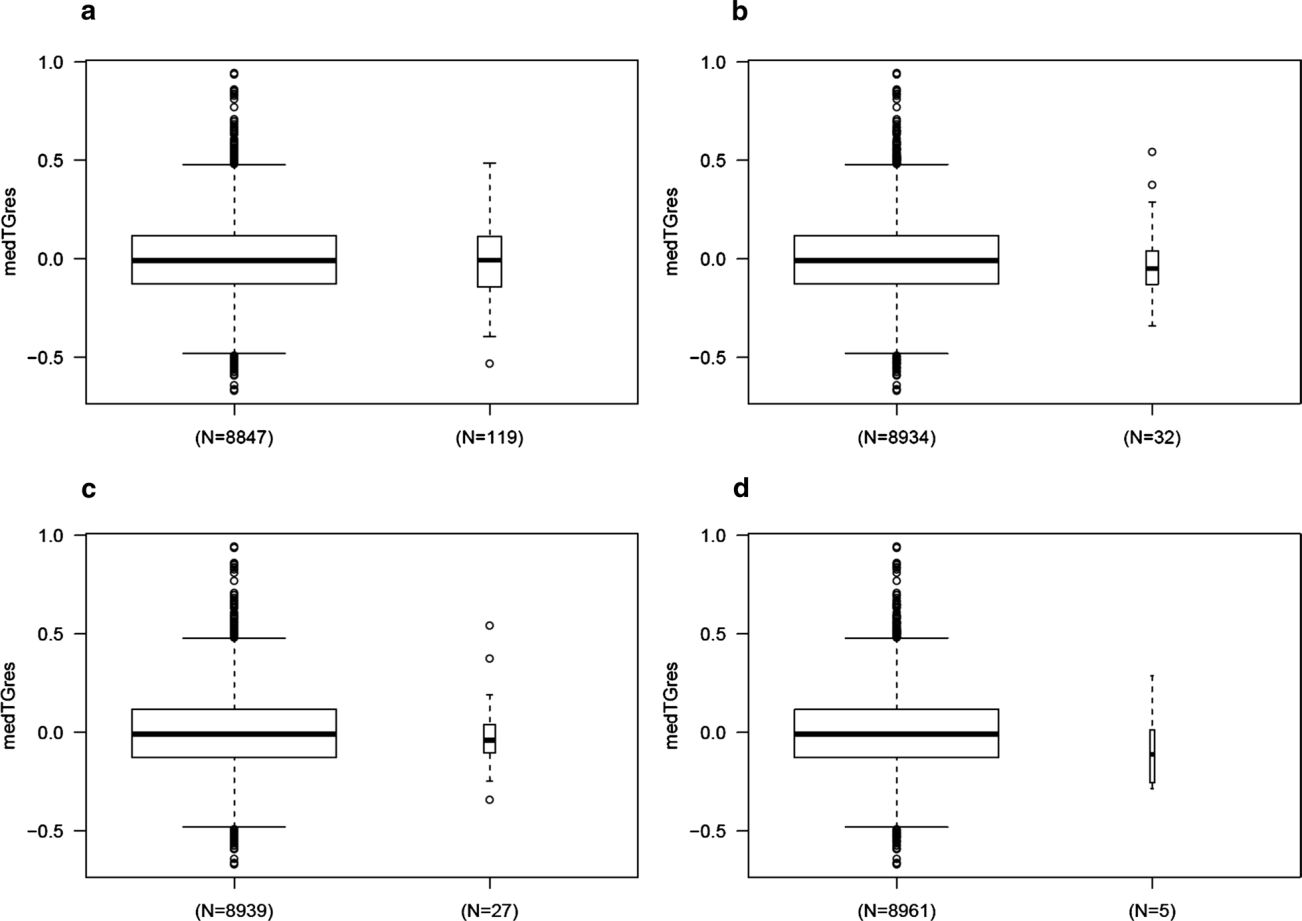
Association between variation at *SLC25A40* and medTGres. **a** rs724665; **b** all rare variation; **c** all rare missense variation; **d** all rare stop gains and frameshifts. Width of the boxplots is proportional to sample size

None of the *SLC25A40* SNVs were significantly associated with medTGres, either separately or in combination (*p* > 0.4, Table 7, Fig. 3). Furthermore, the estimated effect of the variants was negative in all situations (rs724665 *β* = − 0.01; all SNVs excluding rs724665 *β* = − 0.02; all rare missense *β* = − 0.01; rare early terminations *β* = − 0.07). Similar results were observed for maxTGres and for analysis including the EA-only subset.

**Table 7:** Gene tests for *SLC25A40* association with medTGres

Variable	*β*	p
rs724665	−0.01	0.52
Model 2	−0.02	0.49
Model 3	−0.01	0.69
Model 4	−0.07	0.4

Model 2 = all SNVs except for rs724665. Model 3 = all missense SNVs except for rs724665. Model 4 = all early termination SNVs

## Discussion

We replicated association between TG and risk SNVs at *APOE*, *GCKR*, *APOA5* and *APOC3*, with the respective directions of effect as expected. Association between variation at *APOA5* and *APOC3* with TG has been well documented, and our ability to detect these effects supports the reliability of these TG data. Similarly, replication of an association between *APOE* and TG lends further support to the association of *APOE* ε2/3/4 and lipid traits in general. The association between *GCKR* and TG further supports a biological mechanism between diabetes and TG [36]. However, we did not replicate the reported association between TG and risk SNVs at *INSR* and *CETP*. The association between *INSR* rs7248104 and TG was first reported by a large GWAS with a sample size of 176,000 subjects [18]. It is possible that the sample size here is too small to detect any effect. Similarly, the association with *CETP* rs7205804 was discovered in a cohort of 95,000 subjects [37], and therefore it is possible that the sample size here is too small to detect any effect with TG.

Additionally, we failed to replicate an association between putatively deleterious variation at *SLC25A40* and TG. Previous studies relied on linkage in a large family and data from the Exome Sequencing Project (ESP). It is possible that the original signal found in the family is due to another variant in linkage with rs762174003, but unrelated to the function of *SLC25A40*. Further research into this locus may benefit from whole genome sequencing of selected individuals from this family. However, this would not explain why an association was detected in the relatively small sample from the ESP. Results for maximum TG also showed no association.

It is also possible that there is a relationship between *SLC25A40* and TG, but we are not able to detect it in this sample. One major caveat is that we are missing data on treatment for high TG. In our local data set, UWKP, where treatment data were available, we discovered approximately 3% of individuals were being treated for high TG with niacin, fibrates or binders [31]. When we accounted for these treatments in the local data we found evidence supporting an association between TG and rare early termination and conserved missense variation at *SLC25A40* [31]. As the number of participants who harbor such a variant in *SLC25A40* is small (1.7% of our sample), an unaccounted for 3% treatment rate could obscure any signal. Although we used median TG to reduce the effect of unknown treatment, it may have been insufficient as we do not know when treatment would have begun. We did not use the mean as it can be influenced by outliers and TG have a right skewed distribution. Furthermore, this is secondary use of data, which is prone to a high error rate in the data. Although we attempted to clean the data using multiple strategies, it is possible that enough error remained in the data to obscure any true association with *SLC25A40*. In addition, the small numbers of heterozygotes that we observe in this study limit the power of our tests.

## Conclusion

Although we replicated known, strong associations with TG, these data do not replicate the previously reported association between *SL25A40* variation and TG. Larger datasets with more complete data on fasting and medication use may be required to further explore this association. Furthermore, use of secondary data, such as EHR data, needs extensive quality control and would benefit from more comprehensive data extraction methods.

## Data Availability

Data have been posted to dbGaP. Imputed SNV genotype data are available at Study Accession: phs001584.v1.p1 (https://www.ncbi.nlm.nih.gov/projects/gap/cgi-bin/study.cgi?study_id=phs001584.v1.p1). Custom capture sequence data are available at Study Accession: phs001616.v1.p1 (https://www.ncbi.nlm.nih.gov/projects/gap/cgi-bin/study.cgi?study_id=phs001616.v1.p1). Covariate data are available at Dataset Accession: pht009072.v1.p1 (https://www.ncbi.nlm.nih.gov/projects/gap/cgi-bin/dataset.cgi?study_id=phs001584.v1.p1&pht=9072). Median and maximum TG is available upon request.

## Abbreviations

Colu: Columbia University
CVD: Cardiovascular disease
eMERGE: Electronic Medical Records and Genomics
Geis: Geisinger
GERP: Genomic evolutionary rate profiling
Harv: Harvard
HTG: Hypertriglyceridemia
maxTG: Maximum triglycerides
medTG: Median triglycerides
Nwes: Northwestern University
PC: Prinicpal components
TG: Triglycerides
UWKP: University of Washington and Kaiser Permanente Washington
Vand: Vanderbilt
